# Factors associated with linkage to HIV care and TB treatment at community-based HIV testing services in Cape Town, South Africa

**DOI:** 10.1371/journal.pone.0195208

**Published:** 2018-04-02

**Authors:** Sue-Ann Meehan, Rosa Sloot, Heather R. Draper, Pren Naidoo, Ronelle Burger, Nulda Beyers

**Affiliations:** 1 Desmond Tutu TB Centre, Department of Paediatrics and Child Health, Faculty of Medicine and Health Sciences, Stellenbosch University, Cape Town, South Africa; 2 Amsterdam Institute for Global Health and Development, Amsterdam, The Netherlands; 3 Department of Economics, Stellenbosch University, Cape Town, South Africa; The Ohio State University, UNITED STATES

## Abstract

**Background:**

Diagnosing HIV and/or TB is not sufficient; linkage to care and treatment is conditional to reduce the burden of disease. This study aimed to determine factors associated with linkage to HIV care and TB treatment at community-based services in Cape Town, South Africa.

**Methods:**

This retrospective cohort study utilized routinely collected data from clients who utilized stand-alone (fixed site not attached to a health facility) and mobile HIV testing services in eight communities in the City of Cape Town Metropolitan district, between January 2008 and June 2012. Clients were included in the analysis if they were ≥12 years and had a known HIV status. Generalized estimating equations (GEE) logistic regression models were used to assess the association between determinants (sex, age, HIV testing service and co-infection status) and self-reported linkage to HIV care and/or TB treatment.

**Results:**

Linkage to HIV care was 3 738/5 929 (63.1%). Linkage to HIV care was associated with the type of HIV testing service. Clients diagnosed with HIV at mobile services had a significantly reduced odds of linking to HIV care (aOR 0.7 (CI 95%: 0.6–0.8), p<0.001. Linkage to TB treatment was 210/275 (76.4%). Linkage to TB treatment was not associated with sex and service type, but was associated with age. Clients in older age groups were less likely to link to TB treatment compared to clients in the age group 12–24 years (all, p-value<0.05).

**Conclusion:**

A large proportion of clients diagnosed with HIV at mobile services did not link to care. Almost a quarter of clients diagnosed with TB did not link to treatment. Integrated community-based HIV and TB testing services are efficient in diagnosing HIV and TB, but strategies to improve linkage to care are required to control these epidemics.

## Introduction

Globally, the “90-90-90” target has been adopted to end the Acquired Immune Deficiency Syndrome (AIDS) epidemic [1]. South Africa has the largest burden of human immunodeficiency virus (HIV) worldwide, with 7.1 million individuals living with HIV [2]. More than 50% of new tuberculosis (TB) cases are among HIV-infected individuals [3] and TB remains the most common cause of death among HIV-infected adults[4]. The World Health Organization (WHO) emphasises the need to integrate tuberculosis (TB) screening practices into HIV Testing Services (HTS) [5]. The South African Department of Health strongly advocates that HIV services should be used as an entry point for TB screening [6] as South Africa strives toward identifying 90% of individuals with HIV and TB and getting 90% of these individuals started on treatment [6].

In South Africa, utilization of HTS occurs predominantly at public health facilities [7]. Although anyone can test for HIV on their own initiative, public health facilities primarily use a provider-initiated testing strategy, whereby health providers are required to recommend HIV testing to everyone attending health facilities, regardless of whether they have symptoms of HIV[8]. This has been shown to be effective in increasing the number of people who test for HIV [9][10][11]. However, specific subgroups, such as males, are underrepresented in health facilities, [12], hampering access to HIV services for these populations.

Community-based HTS provided on a mobile basis or at stand-alone centres provide a different service offering from the existing public health facilities. Mobile services are provided from a mobile van and ‘pop-up’ tents, set up in public spaces. Stand-alone centres are fixed premises, but not attached to a health facility. Both mobile and stand-alone centres only provide HIV testing and related health services. Public health facilities offer a wider range of health services from fixed sites. Mobile services reach different populations compared to facility-based services; they are more likely to reach males [13], youth (≤25 years) [14] and older individuals (≥31 years) [15][16]. Compared to mobile services, stand-alone services have a higher proportion of individuals who test HIV positive [17] [18].

Linkage to care for individuals diagnosed with HIV and/or TB is essential for individual treatment initiation and for reaching the UNAIDS target. While an estimated 86% of HIV-infected South Africans know their status, only 56% were on antiretroviral therapy (ART) in 2016 [2]. For TB, an estimated 25% of smear-positive TB patients never start treatment [19]. These data emphasise the gap between diagnosis and linkage to care and treatment for both HIV and TB.

Linkage to care is sub-optimal. Linkage to HIV care is estimated at 55% from facility-based provider-initiated testing [20], at 60% from facility-based self-initiated testing [20] [21] and 53% from community-based mobile services [22]. No known published linkage to care data exists at stand-alone services. While community-based services can diagnose HIV and TB among individuals who typically do not access public health facilities [9], there exists limited data around linkage to HIV care and TB treatment from integrated community-based HTS.

In addition, most studies have focused on factors associated with linkage to care at non-integrated services. This study is different because it specifically investigates factors associated with linkage to care at community-based services that have integrated HIV and TB testing. Moreover, in contrast to previous studies, that focused on demographic factors, e [23] [24]; clinical factors, [24]; psychosocial factors, [25], [23][26] [27]; and health service determinants[25], [26], [20], [28] of linkage to care, we investigate the association between service type (mobile and stand-alone services) and linkage to care.

In addition, there is a paucity of data from operational settings and a gap exists for linkage to care data from routinely offered community-based HTS where HIV testing and TB screening services are integrated. To address this gap, this study aimed to quantify linkage to care for HIV and TB and determine factors associated with linkage to HIV care and TB treatment at community-based health services that deliver integrated HIV and TB services in Cape Town, South Africa.

## Methods

### Design and setting

This retrospective cohort study used routinely collected data from clients that attended community-based HTS (stand-alone and mobile) between January 2008 and June 2012 in the City of Cape Town Metropolitan district of the Western Cape Province of South Africa. This district houses 66% of the provincial population. Within this district, HIV prevalence is estimated at 5.2% in the general population [29], and there exists extremely high rates of both HIV-associated and non-HIV-associated TB [30]. Among individuals 15–44 years, HIV and TB are among the leading causes of death [31]. More than 100 primary healthcare facilities offer HIV and TB treatment in the district [32]. This study was conducted within eight communities in this district, all of which are characterised by low socio-economic status [33] and high HIV [31] and TB disease burden [34].

In each of these eight communities, community-based HTS integrating HIV and TB screening, diagnosis and linkage to care was provided at one stand-alone and one mobile service. Stand-alone services were located in shopping malls or residential areas and were fixed in one location for the duration of the study, while mobile services were provided from tents and a caravan (mobile van), strategically set up at various locations within the community, such as transport hubs and along busy thoroughfares. The locations for mobile services were selected on an ad hoc basis and changed regularly over the course of the study period. Anyone could walk in without an appointment and request an HIV test at either of the stand-alone or mobile. Both services were identically staffed by a professional nurse, who provided daily management and clinical services together with an enrolled nurse (provides health care under the supervision of the professional nurses) and three trained lay counsellors, who provided counseling and HIV rapid testing.

All clients self-initiated HTS and underwent pre-test counselling; they were symptomatically screened for TB and consent was taken for an HIV test. All clinical services provided at the mobile and stand-alone were in accordance with Western Cape provincial guidelines. A client was diagnosed HIV positive if both the screening and confirmatory rapid test results were positive. If the rapid screening test was positive, but the rapid confirmatory test was negative (discrepant result), blood was drawn and sent to the National Health Laboratory Service (NHLS) for an enzyme-linked immunosorbent assay (ELISA). The HIV rapid test results were provided during post-test counselling. A client with a discrepant result received the appropriate counselling and was recalled when their ELISA result was available, approximately a week later. Clients diagnosed with HIV were offered a referral letter for HIV care at a public health facility of their choice.

A TB screening tool was used to screen all clients for TB symptoms (cough ≥ 2 weeks, weight loss >1.5 kg, drenching night sweats, fever). Clients who reported one or more symptoms were regarded as presumptive TB cases and two sputum specimens taken at least one hour apart, were sent to the NHLS for TB testing according to the national TB testing algorithm at the time (smear microscopy for all presumptive TB cases and culture for previously treated individuals and smear-negative HIV-infected individuals). A client was diagnosed with TB if the microscopy and/or culture result was positive. All clients with TB were contacted by telephone, recalled to the HIV testing service and provided with a referral letter to a public health facility of their choice to initiate TB treatment.

A health care worker contacted all clients diagnosed with HIV and/or TB by phone, to confirm whether they linked to care or not. Linkage to HIV care and / or TB treatment was defined as self-reported attendance at a public health facility for HIV care / TB treatment within 3 months after being diagnosed at a community-based HIV testing service. If a client reported that they had visited a public health facility for HIV care and/or TB treatment, the healthcare worker recorded that they had linked to care. If clients could not be contacted by telephone, at least three more attempts were made at various times of the day over a 3-month period. Clients that could not be contacted by phone by a health care worker, to confirm whether they linked to care or not, were considered to not have linked to care.

Throughout the study period, all clients diagnosed with TB were eligible for TB treatment [35], but not all clients were eligible for ART. At the beginning of the study period (January 2008) all HIV-positive individuals who had a CD4 count of ≤200 cells/mm^3^ were offered ART at the public health facility [36]. In 2010, the policy changed and ART was offered to HIV-positive individuals with CD4 ≤350 cells/mm^3^ [37] and to all individuals co-infected with TB, irrespective of CD4 count [37]. The study ended prior to the current guidelines (in which all HIV positive individuals are offered ART regardless CD4 count) [38].

### Data collection

The study included data from clients who utilized the stand-alone or mobile services between January 2008 and June 2012. At both services, healthcare workers routinely captured data of each client on paper record forms; including demographic and clinical variables and linkage to care. Each client record had a unique barcode for study purposes. Client records were kept at the HIV testing service for three months before being transported to a central data office. A Microsoft ACCESS 2013 database was specifically designed for this study. Two independent data clerks entered data into two separate datasets, after scanning the client’s unique barcode into the dataset. After comparing the two datasets, a third data clerk validated any differences after referring to the source data (paper forms). This resulted in a final anonymized dataset, with no individual identifiers. All clients ≥12 years, who had an HIV test done and a documented HIV test result, were included in the analysis.

### Statistical analysis

This study had two primary outcomes of interest: 1) linkage to HIV care among clients diagnosed with HIV at HTS; 2) linkage to TB treatment among clients diagnosed with TB at HTS. Determinants (sex, age, HIV testing service and co-infection status) were identified using logistic regression. Generalised estimating equations (GEE) were incorporated to control for correlated data (two HTS services were located within the same community).All variables were included in the multivariate analysis, irrespective of their association with the outcome in univariate analysis. The level of significance in all analyses was p<0.05. Analyses was completed in Stata (StataCorp. 2015. Stata Statistical Software: Release 14. College Station, Texas, USA: StatCorp LP).

### Ethics approval

The Health Research Ethics Committee of Stellenbosch University (N08/10/307) approved the study, which was conducted according to the guiding principles within the Declaration of Helsinki. All clients who underwent an HIV rapid test provided written consent on their client record form. No incentives were provided.

## Results

Overall 79 545 clients, with a known HIV test result were included in the study, of which 39 142 were males. The median age of all clients was 29 years (IQR 22–41).

### Linkage to HIV care

**Fig 1:**
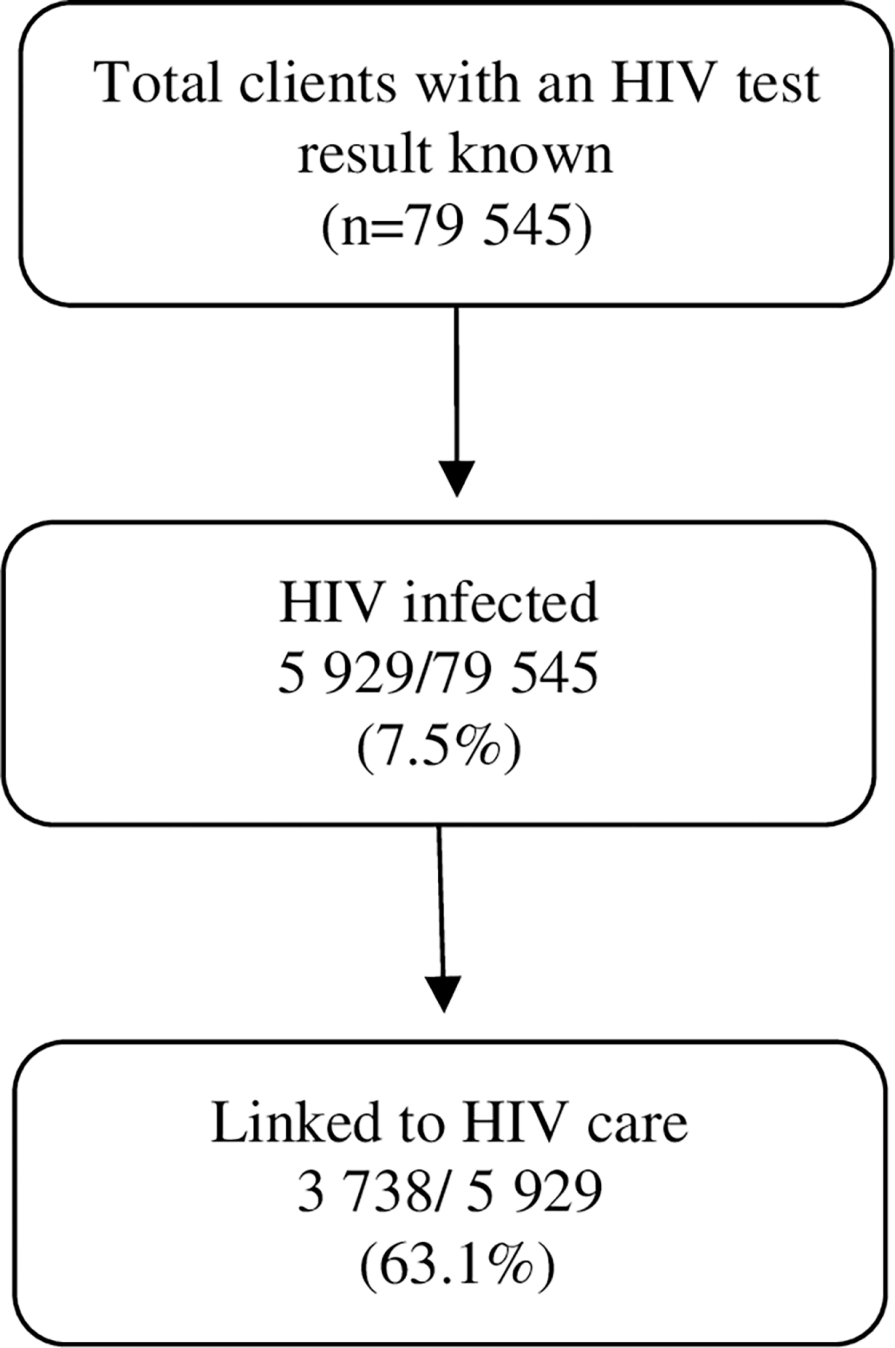
Linkage to HIV care for clients with known HIV status at community-based HIV testing services in the City of Cape Town Metropolitan district, Western Cape, South Africa.

Of the 79 545 clients who had a known HIV test result, 5 929 (7.5%) were diagnosed with HIV (1 870 at stand-alone and 4 059 at mobile). Of these 3 738 (63.1%) were linked to HIV care (1 311 at stand-alone and 2 427 at mobile). See Fig 1. Among males, 1 388/2 159 (64.3%) linked to HIV care. The median age of clients linked to HIV care was 30 years (IQR: 25–37). Among clients co-infected with TB, 59/86 (68.6% linked to HIV care. At the stand-alone service, linkage to HIV care was significantly higher as compared to linkage to HIV care at the mobile service (70.1% vs 59.8%, p<0.001) (Table 1).

**Table 1:** Characteristics of clients diagnosed with HIV and TB between 2008 and 2012 at integrated community-based HIV testing services in the City of Cape Town Metropolitan district, by linkage to HIV care and TB treatment.

	Clients diagnosed with HIV			Clients diagnosed with TB		
	Linked to HIV care (n, %)	Not linked to HIV care (n, %)	p-value*	Linked to TB treatment (n, %)	Not linked to TB treatment (n, %)	p-value*
Total	3,738 (63.1)	2,191 (36.9)		210 (76.4)	65 (23.6)	
**Sex**						
Male	1,388 (64.3)	771 (35.7)	0.133	121 (73.3)	44 (26.6)	0.147
Female	2,350 (62.3)	1,420 (37.7)		89 (80.9)	21 (19.1)	
**Age (years)**						
Median (IQR)	30 (25–37)	30 (25–37)	0.117**	29 (25–41)	36 (27–45)	0.007**
12–24	828 (60.8)	533 (39.2)	0.355	51 (87.9)	7 (12.1)	0.086
25–34	1,612 (64.0)	905 (36.0)		82 (78.1)	23 (21.9)	
35–44	868 (63.4)	502 (36.6)		35 (70.0)	15 (30.0)	
≥45	379 (63.6)	217 (36.4)		38 (67.9)	18 (32.1)	
Unknown	51 (60.0)	34 (40.0)		4 (66.7)	2 (33.3)	
**HIV testing service**						
Stand-alone	1,311 (70.1)	559 (29.9)	<0.001	121 (78.1)	34 (21.9)	0.451
Mobile	2,427 (59.8)	1,632 (40.2)		89 (74.2)	31 (25.8)	
**HIV/TB co-infection**						
No	3,679 (63.0)	2,164 (37.0)	0.282	137 (72.5)	52 (27.5)	0.025
Yes	59 (68.6)	27 (31.4)			73 (84.9)	13 (15.1)	

* Chi squared test, unless otherwise stated.

**Mann Whitney U-test.

IQR: Interquartile range.

Compared to clients diagnosed with HIV at stand-alone services, clients diagnosed with HIV at mobile services had a significantly reduced odds of linking to HIV care (aOR 0.7 (CI 95%: 0.6–0.8), p<0.001 (Table 2). Linkage to HIV care was not associated with sex, age or TB co-infection. Multivariable analysis in S1 Table shows that linkage to HIV care at both the stand-alone and mobile modalities were not associated with sex, age or TB co-infection.

**Table 2:** Factors associated with to linkage to HIV care and TB treatment among adolescents and adults between 2008 and 2012 in the City of Cape Town Metropolitan district, South Africa.

	Linkage to HIV care		Linkage to TB treatment	
	Adjusted OR (95%CI)	p-value	Adjusted OR (95%CI)	p-value
**Sex**				
Male	1		1	
Female	0.9 (0.9–1.1)	0.658	1.2 (0.7–2.0)	0.426
**Age (years)**				
12–24	1		1	
25–34	1.1 (0.9–1.3)	0.064	0.5 (0.2–0.9)	0.046
35–44	1.1 (0.9–1.3)	0.222	0.3 (0.1–0.7)	0.007
≥45	1.1 (0.9–1.4)	0.181	0.4 (0.2–0.8)	0.019
Unknown	1.1 (0.7–1.8)	0.681	0.4 (0.1–2.4)	0.327
**HIV testing service**				
Stand-alone	1	<0.001	1	0.798
Mobile	0.7 (0.6–0.8)		0.9 (0.6–1.6)	
**HIV/TB co-infection**				
No	1		1	
Yes	1.2 (0.7–1.8)	0.555	1.3 (0.7–2.3)	0.334

OR: odds ratio.

### Linkage to TB treatment

**Fig 2:**
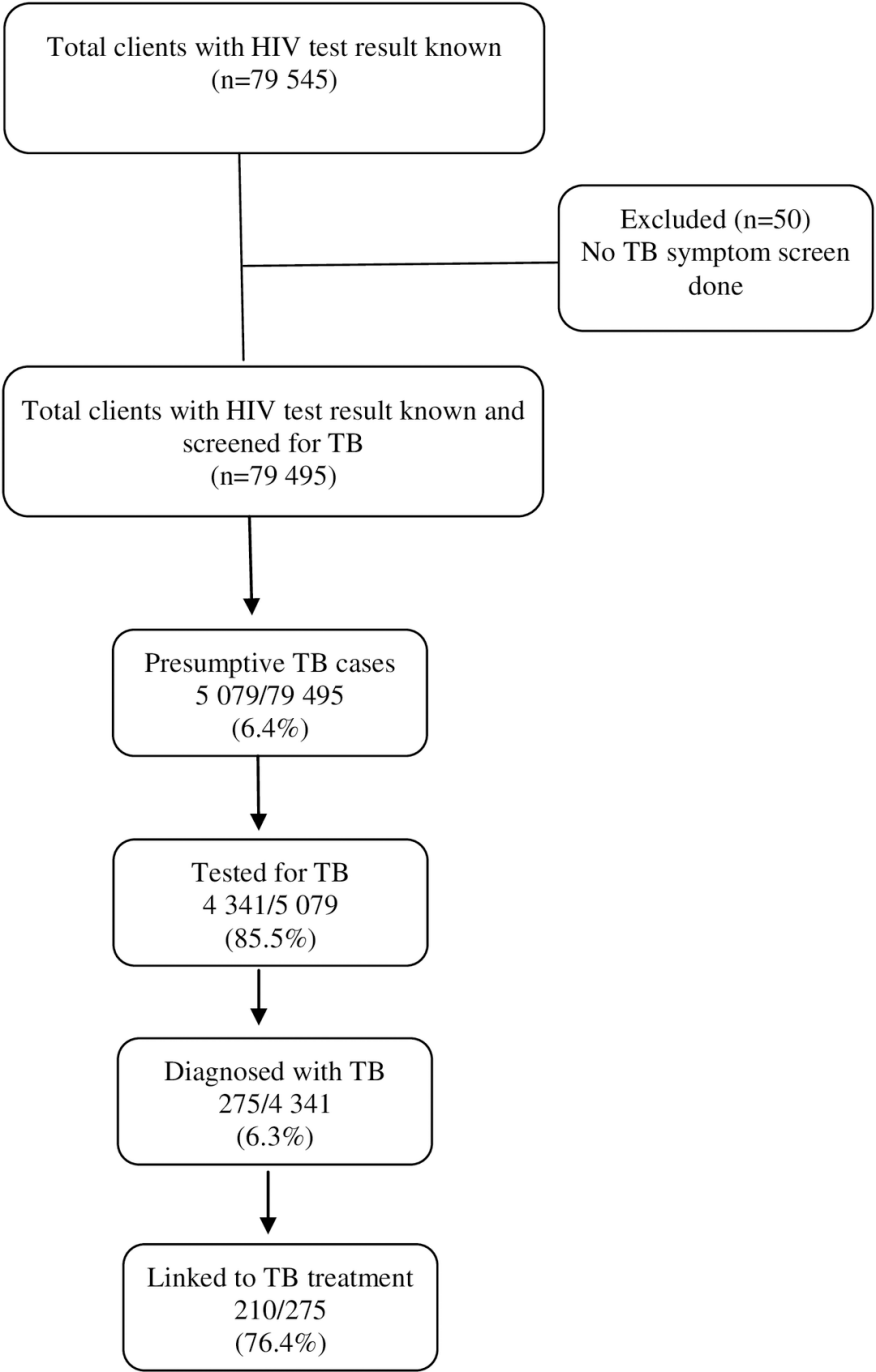
Linkage to TB treatment for clients with known HIV status at integrated community-based HIV testing services in the City of Cape Town Metropolitan district, Western Cape, South Africa.

Of the 79 545 clients with known HIV status, 50 were excluded from the analysis as no TB symptom screening was done. Of the 79 495 clients screened for TB, 5 079 (6.4%) were presumptive TB cases, of which 4 341 (85.5%) were tested for TB. Of those tested, 275 (6.33%) were diagnosed with TB (155 at stand-alone and 120 at mobile). The majority (76.4%) linked to TB treatment (121 at stand-alone and 89 at mobile). See Fig 2.

Among males, 121/165 (73.3%) initiated TB treatment. Clients who initiated TB treatment were younger (median age 29 years, IQR: 25–41) compared to those who did not initiate TB treatment (median age 36 years, 95% CI: 27–45) (p≤0.007). Among clients co-infected with HIV, linkage to TB treatment was higher as compared to linkage among those without co-infection (84.9% vs 72.5%, p = 0.025). See Table 1.

Table 2 shows that linkage to TB treatment was associated with age. Clients in the age groups 25–34 (p = 0.046), 35–44 (p = 0.007) and ≥45 years (p = 0.019) were less likely to link to TB treatment compared to clients in the age group 12–24 years. Linkage to TB treatment was not associated with sex, HIV testing service or HIV co-infection. At the stand-alone, linkage to TB treatment was not associated with sex, age or HIV co-infection. At mobile, clients in the age groups 25–34, 35–44 and ≥45 years were less likely to commence TB treatment compared to clients in the age group 12–24 years (all, p-value<0.02). See S1 Table.

## Discussion

This study quantified linkage to HIV care and TB treatment and determined factors associated with HIV care and TB treatment at an integrated community-based HTS, offering HIV testing and TB screening and testing from stand-alone and mobile services. Linkage to HIV care was 63.1% and was associated with service type (at the stand-alone service, linkage to HIV care was significantly higher as compared to linkage to HIV care at the mobile service). Of those diagnosed with TB, 76.4% linked to TB treatment, which was associated with age (clients who initiated TB treatment were younger compared to those who did not initiate TB treatment).

Linkage to HIV care in this study is higher than what has been reported from mobile services in other Cape Town studies (51%-53%) [22][24]. Studies use different definitions of linkage to care, making comparison difficult.

Our study found that the type of HIV testing service (mobile or stand-alone) was associated with linkage to HIV care. This differs from a study in Swaziland, that found that the type of HIV testing service (home-based compared to mobile) was not associated with linkage to HIV care [39]. In our study, mobile services diagnosed and linked more clients to HIV care than the stand-alone service (2 427 clients at mobile and 1 311 clients at stand-alone were linked to care), indicating the important role that mobile HTS play in diagnosing and linking large numbers of individuals with HIV. However, clients diagnosed with HIV at mobile services were significantly less likely to link to care (59.8%) as compared to those diagnosed at stand-alone services (70.1%). This finding emphasises the need for improved linkage to HIV care interventions and suggests that efforts to improve linkage to care from mobile could have a significant impact on increasing linkage to care overall. Interventions that provide client support after diagnosis, including additional counselling and accompanying the client to the health facility may improve linkage to HIV care. Future operational research studies should compare different interventions at mobile services to determine more efficient linkage to care.

Awareness of symptoms of disease has been associated with voluntary uptake of counselling and testing [40][41]. Awareness of disease may also play a role in linkage to care. Clients who accessed a stand-alone service for an HIV test may have identified signs or symptoms of disease and actively sought an HIV test. Those who tested at the mobile may have taken the immediate opportunity to test that a mobile service offers [42] and may not have been aware of any signs or symptoms of disease. The presumption that clients diagnosed at mobile were not driven to test due to their own awareness of symptoms may have delayed them linking to care. Studies have shown that individuals who are feeling “well” i.e. have higher CD4 counts [24], are asymptomatic [27], have no TB symptoms [24] are less likely to link to HIV care. Future research is required to investigate factors associated with linkage to care from community-based services to provide an empirical basis for designing interventions aimed at improving linkage to care, in particular from mobile HTS.

Sex, age and TB co-infection were not associated with linkage to HIV care. This concurs with a Cape Town study that showed age and sex did not predict linkage to care for individuals who self-initiated an HIV test at public health facilities [21], but differs from another study that found older individuals were more likely to link to care from home-based services [23].

Overall, the majority of clients diagnosed with TB linked to treatment. This finding is higher than what was reported in another Cape Town study where 57% of individuals diagnosed with TB, linked to care from a mobile service [22]. Our finding is similar to that found in individuals diagnosed with TB at public health facilities [19]. Although the majority of clients diagnosed with pulmonary TB in our study linked to Tb treatment, almost a quarter did not. This has serious public health implications as these individuals continue to spread TB in their communities.

In our study, of clients diagnosed with TB, 76.4% linked to TB treatment compared to 63.1% of HIV-infected clients who linked to HIV care. We hypothesise that; (i) there were a smaller number of clients diagnosed with TB (275) compared to the number diagnosed with HIV (5 929) and healthcare workers may have found this more manageable, (ii) as pulmonary TB is infectious, healthcare workers may have made more of an effort to link these clients to care, or (iii) TB treatment was available to everyone diagnosed with TB whereas antiretroviral therapy was only available to individuals who met the eligibility criteria at the time. As point-of-care CD4 testing was not done in this study, clients would have been unaware of their eligibility for ART until they linked to HIV care. Further research is needed to test these assumptions to better understand how linkage to care and treatment may be different for those diagnosed with HIV and TB.

Linkage to TB treatment was associated with age. Compared to clients aged 12–24 years, clients in older age groups were less likely to link to TB treatment. Younger clients may have been easier to follow up, especially if they were still in school or this may have been their first TB episode and they may have been more motivated to link to treatment than older clients, who may have experienced previous TB treatment. More research is needed to better understand the association between age and linkage to TB treatment. Sex, HIV testing service and HIV co-infection were not associated with linkage to TB treatment. There is a lack of literature around factors associated with linkage to TB treatment from community-based HTS and more work is needed in this area.

In order to reach the ‘90-90-90’ goals and bring the dual TB and HIV epidemics under control, improved linkage to care and treatment is essential. Although our study showed higher rates of linkage to care compared to other studies from Cape Town, these remain suboptimal. In our study setting, general messaging supported TB as a curable disease [43][44] and HIV as a lifelong chronic disease [45] and TB and HIV care and treatment was freely available from a multitude of primary health care facilities. We therefore speculate that other factors, apart from promoting the benefits of treatment and having treatment services available free of charge, are associated with linkage to care and treatment. Community-based services could consider establishing an effective referral network, accompanying the patient to the health facility [46] and providing on-going follow-up to improve linkage to HIV care and treatment.

ART is currently available for all people living with HIV in South Africa, irrespective of CD4 count (universal test and treat (UTT)) [6][38]. We speculate that linkage to HIV care will not necessarily improve as UTT is rolled out. We hypothesise that initial awareness of signs or symptoms of disease may play a larger role in linkage to care and treatment than demographic (e.g. sex and age), clinical (e.g. diagnosed disease or co-infection) or health service factors (referral letter). Future studies should determine the impact of UTT on linkage to care from community-based HTS.

A major strength of this study is that it incorporates a large number of client records that were routinely collected from community-based HTS modalities (8 stand-alone centres and 8 mobile services), implemented locally, to generate knowledge around linkage to HIV care and TB treatment. This study adds to the limited body of literature that describes integration of TB services into community-based HIV testing and linkage to care and treatment from community-based HTS in South Africa. Previous research has focused on linkage within HIV or TB services, but not an integrated service. Secondly, this study identified the need for specific linkage to HIV care interventions from mobile HTS.

The main limitation is that linkage to care and treatment was self-reported. It was not possible to confirm linkage to care against health facility records. Acknowledging that it takes time for clients to come to terms with an HIV positive diagnosis, some clients may have linked to care after the 3-month period. Potentially, linkage to care reported in this study may be underestimated, but balanced by over-reporting of socially desirable answers given over the phone by clients. Secondly, the routine data for this study was collected during a period when eligibility criteria for ART were based on a CD4 threshold. No data on CD4 count was collected and therefore it was not possible to determine linkage to care trends over the study period according to eligibility criteria of new guidelines. Future studies should evaluate linkage to care trends over time and the influence of eligibility criteria. Thirdly, only a few variables were available to be evaluated because the study used routinely collected data. This makes the study limited in terms of its findings. In addition, the results are only generalizable to similar peri-urban areas; future research should determine factors associated with linkage to care for community-based services in rural settings.

## Conclusion

In order to reach the ‘90-90-90’ target and control dual HIV and TB epidemics in South Africa, improved linkage to care is vital. A large proportion of clients diagnosed with HIV at mobile services did not link to care and almost a quarter of clients diagnosed with TB did not link to treatment. Integrated community-based HIIV testing services can diagnose HIV and TB, but improved linkage to care strategies are vital. Future studies should investigate interventions that provide extended client support to facilitate improved linkage to care in similar HIV and TB high-burden settings for improved impact on public health.

## Supporting information

S1 Table Factors associated with linkage to HIV care and TB treatment among adolescents and adults between 2008 and 2012 in the Cape Town Metropolitan district, South Africa, by HIV testing service. (DOCX)
