# Supplementary material for: Factors associated with linkage to HIV care and TB treatment at community-based HIV testing services in Cape Town, South Africa
Source: PLoS One. 2018 Apr 2;13(4):e0195208. doi: 10.1371/journal.pone.0195208 (PMC5880394; doi:10.1371/journal.pone.0195208)
Supplement: S1 Table — (DOCX) [file pone.0195208.s001.docx]

**S1 Table: Factors associated with linkage to HIV care and TB treatment among adolescents and adults between 2008 and 2012 in the Cape Town Metropolitan district, South Africa, by HIV testing service**

|  | **Linkage to HIV care** | | | |  | **Linkage to TB treatment** | | | |
| --- | --- | --- | --- | --- | --- | --- | --- | --- | --- |
|  | **Stand-alone** | | **Mobile** | |  | **Stand-alone** | | **Mobile** | |
|  | Adjusted OR (95%CI) | p-value | Adjusted OR (95%CI) | p-value |  | Adjusted OR (95%CI) | p-value | Adjusted OR (95%CI) | p-value |
| **Sex** |  |  |  |  |  |  |  |  |  |
| Male | 1 |  | 1 |  |  | 1 |  | 1 |  |
| Female | 1.0 (0.8-1.2) | 0.914 | 0.9 (0.8-1.1) | 0.393 |  | 1.1 (0.6-2.1) | 0.720 | 1.8 (0.9-3.7) | 0.115 |
| **Age (years)** |  |  |  |  |  |  |  |  |  |
| 12-24 | 1 |  | 1 |  |  | 1 |  | 1 |  |
| 25-34 | 1.2 (0.9-1.5) | 0.228 | 1.1 (0.9-1.3) | 0.216 |  | 1.2 (0.5-2.8) | 0.743 | 0.2 (0.1-0.7) | 0.010 |
| 35-44 | 1.3 (0.9-1.7) | 0.110 | 1.0 (0.9-1.3) | 0.722 |  | 0.9 (0.3-2.4) | 0.760 | 0.2 (0.1-0.5) | 0.003 |
| ≥45 | 1.2 (0.8-1.8) | 0.394 | 1.1 (0.9-1.4) | 0.340 |  | 0.8 (0.3-2.2) | 0.619 | 0.2 (0.1-0.7) | 0.008 |
| Unknown | 0.8 (0.3-2.1) | 0.675 | 1.2 (0.7-2.0) | 0.542 |  | 0.5 (0.1-3.7) | 0.471 | N.A. | N.A. |
| **HIV/TB co-infection** |  |  |  |  |  |  |  |  |  |
| No | 1 |  | 1 |  |  | 1 |  | 1 |  |
| Yes | 1.1 (0.6-1.9) | 0.768 | 1.2 (0.6-2.7) | 0.617 |  | 1.2 (0.6-2.4) | 0.595 | 1.1 (0.5-2.6) | 0.834 |
